# Docosanoid signaling modulates corneal nerve regeneration: effect on tear secretion, wound healing, and neuropathic pain

**DOI:** 10.1194/jlr.TR120000954

**Published:** 2021-02-06

**Authors:** Thang L. Pham, Haydee E.P. Bazan

**Affiliations:** 1 Neuroscience Center of Excellence and Department of Ophthalmology, School of Medicine, Louisiana State University Health New Orleans, New Orleans, LA, USA

**Keywords:** cell signaling, gene expression, lipoxygenase, omega 3 fatty acids, phospholipase A2, dry eye, stereoisomer of resolvin D6, neuroprotectin D1, docosahexaenoic acid, pigment epithelium-derived factor, BDNF, brain-derived neurotrophic factor, COX, cyclooxygenase, DE, dry eye, DED, dry eye disease, HDHA, hydroxy-DHA, LOX, lipoxygenase, NGF, nerve growth factor, NPD1, neuroprotectin D1, PEDF, pigment epithelium-derived factor, PEDF-R, pigment epithelium-derived factor receptor, PRK, photorefractive keratectomy, RvD6, resolvin D6, SP, substance P, TG, trigeminal ganglia

## Abstract

The cornea is densely innervated, mainly by sensory nerves of the ophthalmic branch of the trigeminal ganglia (TG). These nerves are important to maintain corneal homeostasis, and nerve damage can lead to a decrease in wound healing, an increase in corneal ulceration and dry eye disease (DED), and neuropathic pain. Pathologies, such as diabetes, aging, viral and bacterial infection, as well as prolonged use of contact lenses and surgeries to correct vision can produce nerve damage. There are no effective therapies to alleviate DED (a multifunctional disease) and several clinical trials using ω-3 supplementation show unclear and sometimes negative results. Using animal models of corneal nerve damage, we show that treating corneas with pigment epithelium-derived factor plus DHA increases nerve regeneration, wound healing, and tear secretion. The mechanism involves the activation of a calcium-independent phospholipase A2 that releases the incorporated DHA from phospholipids and enhances the synthesis of the docosanoids, neuroprotectin D1 (NPD1) and a new resolvin stereoisomer, resolvin D6i (RvD6i). NPD1 stimulates the synthesis of brain-derived neurotrophic factor, nerve growth factor, and semaphorin 7A. RvD6i treatment of injured corneas modulates gene expression in the TG resulting in enhanced neurogenesis, decreased neuropathic pain, and increased sensitivity. Taken together, these results represent a promising therapeutic option to reestablish the homeostasis of the cornea.

## Cornea anatomy

**Figure:**
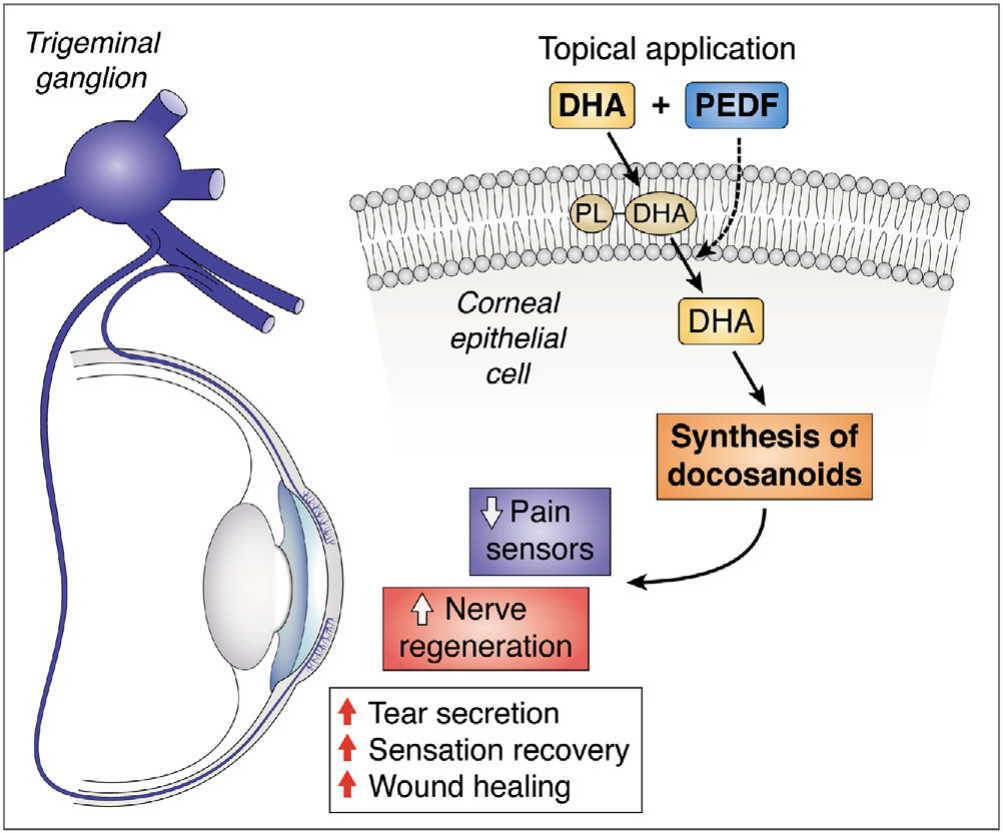

**Fig. 1:**
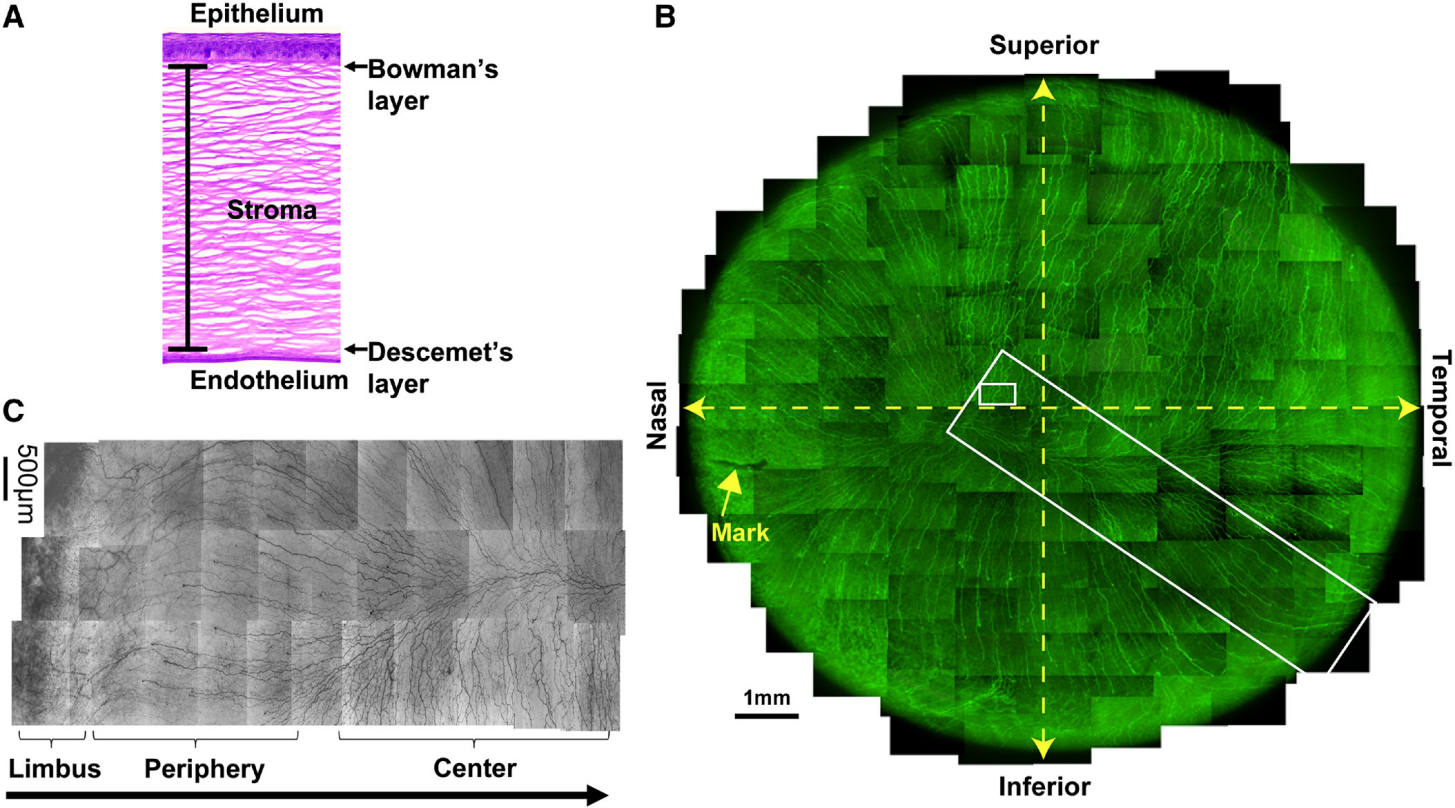
Corneal structure and innervation. A: The anatomy of human cornea after hematoxylin and eosin histological stain. All five layers are shown: epithelium, Bowman’s layer, stroma, Descemet’s layer, and endothelium. B: Whole mount view of complete human corneal epithelial nerve network obtained from the left eye of a 45-year-old male donor. C: Detailed course of epithelial nerve bundles running from the periphery to the convergence at the center of the cornea (B and C are reproduced with permission from Elsevier, Ref. 5).

The transparent cornea accounts for 70% of the refractive power of the human eye by allowing light to pass through and be projected to the retina. In addition, the cornea also provides an important barrier to regulate immune response and to prevent pathogens from entering the ocular globe. Anatomically, the cornea can be divided into five sublayers: epithelium, Bowman’s layer, stroma or substantia propria, Descemet’s membrane, and endothelium (1, 2) (Fig. 1A).

The epithelium consists of five to seven layers of nonkeratinized squamous epithelial cells, which are classified into three morphological cell types: superficial epithelial cells, intermediate wing cells, and the innermost basal epithelial cells with high rates of proliferation (2). The epithelial cells are connected by tight junctions that block the passage of foreign materials, such as dust, water, and bacteria, into the eye and provide a smooth surface that absorbs oxygen and cell nutrients. Moreover, the outermost layer of the epithelium is in contact with the tear film, which facilitates the moistness maintenance of the ocular surface that protects against damage from drying [dry eye (DE)]. Corneal epithelial cells regularly undergo a “turnover” with movement of stem cells from the limbal epithelium to the basal layer. These basal cells move toward the surface to generate two to three layers of wing cells and then begin terminal differentiation and desquamation. On average, the turnover time of human corneal epithelial cells is between 7 and 10 days (3).

The Bowman’s layer is a thin acellular layer that separates the epithelium from the stroma. It mainly contains collagen IV and laminin. The organization of these proteins is important to maintain the transparency of the tissue.

The stroma layer is built up by quiescent keratocytes and a well-organized extracellular matrix composed primarily of highly ordered collagen type 1 fibrils, called lamella, and proteoglycans, and also constitutes the largest portion of the cornea (about 90% of corneal thickness). The stroma provides structural support to the cornea as well as transparency by facilitating the passage of light through collagen fibrils in a manner that prevents scattering. Keratocytes (the flat cells situated between collagen fibers) are the main cell residents of corneal stroma.

The Descemet’s membrane is an acellular thin layer synthesized by the endothelium that is composed of fibronectin, laminin, and collagen IV and VII as well as proteoglycans. Damage to the Descemet’s membrane produces corneal edema and loss of vision.

The last layer of the cornea is the endothelium, which is in contact with the aqueous humor. It is a monolayer of cells responsible for pumping fluid to regulate corneal stromal dehydration. Without endothelial pumps, there will be stroma edema, which produces opacity and decrease in vision. The human corneal endothelial cells have a very low capacity for proliferation, resulting in age-related reduction in cell density.

An important characteristic of the cornea is its dense innervation (Fig. 1B). Most corneal nerve fibers are sensory in origin and derived mostly from neurons of the ophthalmic branch of the trigeminal ganglia (TG) (4, 5, 6). Anatomically, the corneal nerve network originates when stromal nerves enter the corneal sclera limbus in a radial fashion. To maintain corneal transparency, the arriving nerves lose their myelin sheaths and are surrounded by Schwann cells alone. In the stroma, the thick branches are divided into smaller nerve branches. Most of the branches penetrate the Bowman’s layer in the periphery and run to the center of the epithelium to form the epithelial nerve network (Fig. 1C), giving life to a dense network of nerve terminals.

Corneal nerves stimulate tear secretion and blinking to maintain the integrity of the ocular surface (7). Alterations in corneal innervation occur in aging, diabetes, immunological diseases, such as rheumatoid arthritis and Sjögren’s syndrome, viral and bacterial infection, prolonged use of contact lenses, and refractive surgeries, such as laser in situ keratomileusis (LASIK) and photorefractive keratectomy (PRK) (10, 11, 12, 13, 8, 9). Complications from nerve damage diminish sensitivity, decrease tear secretion and blinking, and as a consequence, result in DE disease (DED) that produces neuropathic pain and corneal ulceration in severe cases. Due to the abundance of sensory nerves, the cornea is also a potent generator of pain in the human body.

## PEDF+DHA treatment for cornea-related damage: discovery of a resolvin D6 stereoisomer

As mentioned, after damage, corneal nerve density slowly and incompletely recovered with decrease in sensitivity and DE symptoms. Early studies from our laboratory have shown that application of nerve growth factor (NGF) in conjunction with the ω-3 fatty acid DHA results in faster recovery of corneal nerve density after experimental PRK in rabbits (14). At that time, we proposed that the mechanisms could be mediated by the DHA-derived lipid mediator neuroprotectin D1 (NPD1), a docosanoid with potent anti-inflammatory and neuroprotective actions (15). Synthesis of NPD1 in retinal pigment epithelial cells is stimulated by several growth factors with pigment epithelium-derived factor (PEDF) being 10 times more potent than NGF (16). PEDF is a broad-acting neurotrophic and neuroprotective factor that regulates processes associated with angiogenesis, neuronal cell survival, and cell differentiation (17) and is released from corneal epithelium after injury (18). Posterior studies have shown that treatment with PEDF+DHA decreases inflammation and stimulates corneal wound healing and nerve regeneration in rabbit and mouse cornea models of experimental surgery, as well as in pathologies like diabetes and herpes simplex virus infection (19, 20, 21, 22, 23). It is important to mention that the action requires treatment with both PEDF and DHA (19). A 44-amino acid fragment of PEDF has neuroprotective activity, while an adjacent 34-amino acid peptide has anti-angiogenic activity (24, 25). Comparing the effect of the two peptides with the whole PEDF protein plus DHA in a rabbit model of corneal stroma dissection, we found that, unlike 34-mer-PEDF, 44 mer-PEDF+DHA decreases inflammation and increases tear secretion and corneal sensitivity and also promotes regeneration of corneal nerves by activating a PEDF receptor (PEDF-R) (21). This transmembrane receptor is expressed in the cornea and has calcium-independent phospholipase A2 (iPLAζ) activity (26, 27) that releases DHA, which is enriched in the sn-2 position of membrane phospholipids by DHA supplementation.

Early studies on calf corneas identified PC, PE, and sphingomyelin as the main phospholipids in the tissue (28). Among these phospholipids, PC is the most abundant with the highest content in the epithelium. Similar observations were reported in human (29) and rabbit corneas (30). In the rabbit, oleic acid (18:1) is the dominant fatty acid esterified in phospholipids in all of the corneal layers (about 50% of total fatty acids in phospholipids) followed by palmitic acid (16:0), which comprises about 16–18%. With respect to the PUFAs esterified in phospholipids, the higher percentage (about 9% of total fatty acids) corresponds to AA, while the percentage of EPA and DHA esterified in phospholipids is much lower (around 1.6% of total fatty acids) (30).

**Fig. 2:**
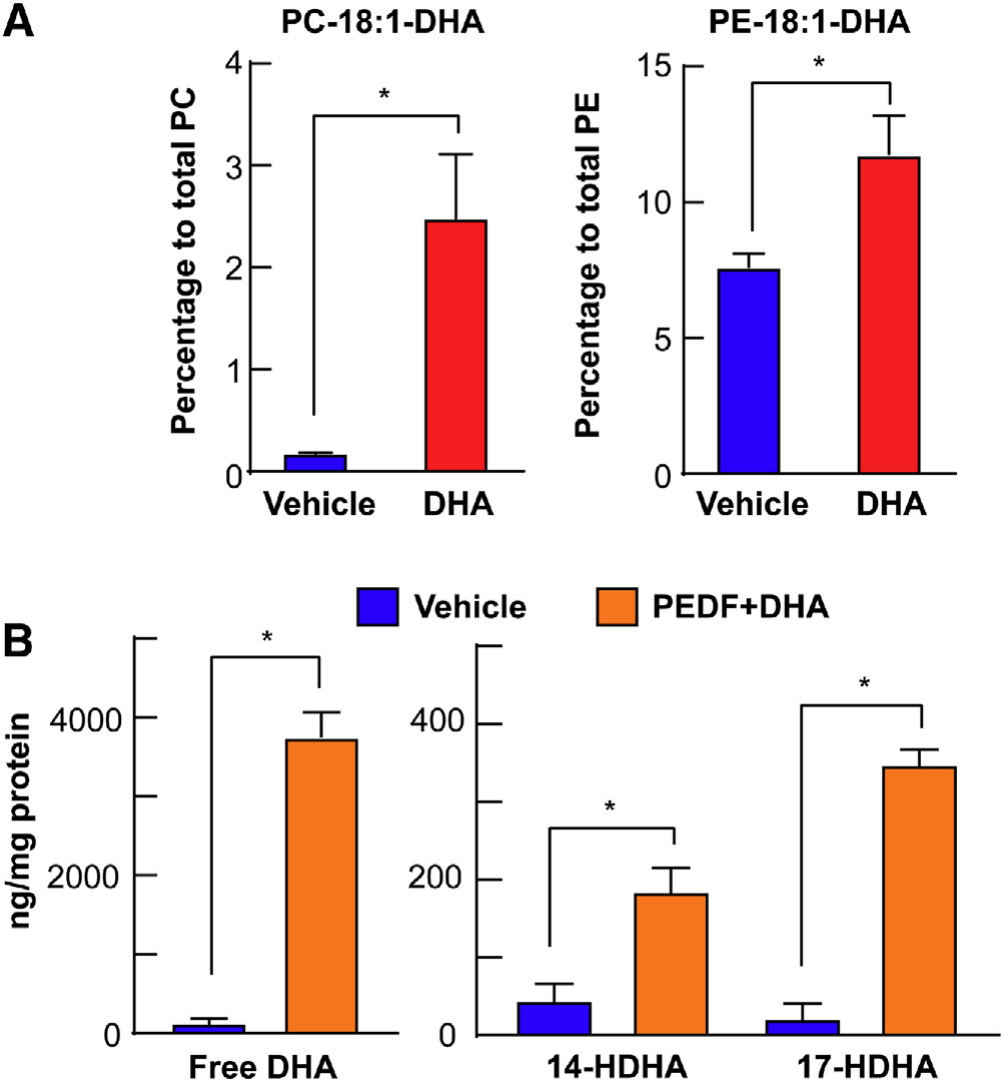
Incorporation of DHA into PC and PE after 1 h of DHA topical treatment to corneas of mice with damaged stromal nerves. A: Mouse corneas were injured and topically treated with DHA for 1 h and then lipids extracted and analyzed by LC-MS/MS (27). The proportion of PC and PE containing oleic acid (18:1) in the sn-1 position and DHA in the sn-2 position. PE was more enriched in DHA than PC. B: Release of DHA and synthesis of the monohydroxy-DHA derivatives after corneal injury and topical treatment with PEDF+DHA for 3 h. Corneal lipid profiles were analyzed by MS-based lipidomic analysis. ∗*P* < 0.05 with the *t* test statistical analysis to compare two groups at 95% of the confidence level.

DHA topical treatment of mouse corneas, in which stromal nerves had been damaged, produced a rapid incorporation of the fatty acid in PC and PE molecular species containing 18:1-DHA (27), demonstrating that the addition of the PUFAs created a significant enrichment of DHA in the lipid membrane composition (Fig. 2A).

Tissue damage activates phospholipase A2 that releases PUFAs, such as AA, EPA, and DHA, from the sn-2 position (31, 32). Several early studies from our laboratory and others have demonstrated that the cornea responds to injury, increasing the synthesis of prostaglandins by activation of cyclooxygenase (COX)-2 (33, 34, 35, 36) and HETEs and lipoxin A4 (LxA4) by activation of lipoxygenases (LOXs) (37, 38, 39). Because the concentration of DHA in membrane lipids is very low (Fig. 2A) (30), we found that the addition of DHA to the corneas treated with PEDF was important to increasing the synthesis of lipid derivatives of DHA (docosanoids) with strong anti-inflammatory properties (19, 40, 41). Therefore, activating the calcium-independent phospholipase A2 (iPLA2ζ) of the PEDF-R by treating the corneas with PEDF+DHA leads to a more than 3,000-fold increase of free DHA released from the cornea (Fig. 2B).

Free DHA is then the substrate for the synthesis of 14- and 17-hydroperoxy-DHA that are rapidly converted in the more stable hydroxy-DHA (HDHA) derivatives (Fig. 2B). These results confirmed our hypothesis that PEDF+DHA treatment stimulates the formation of docosanoids derived from DHA.

**Fig. 3:**
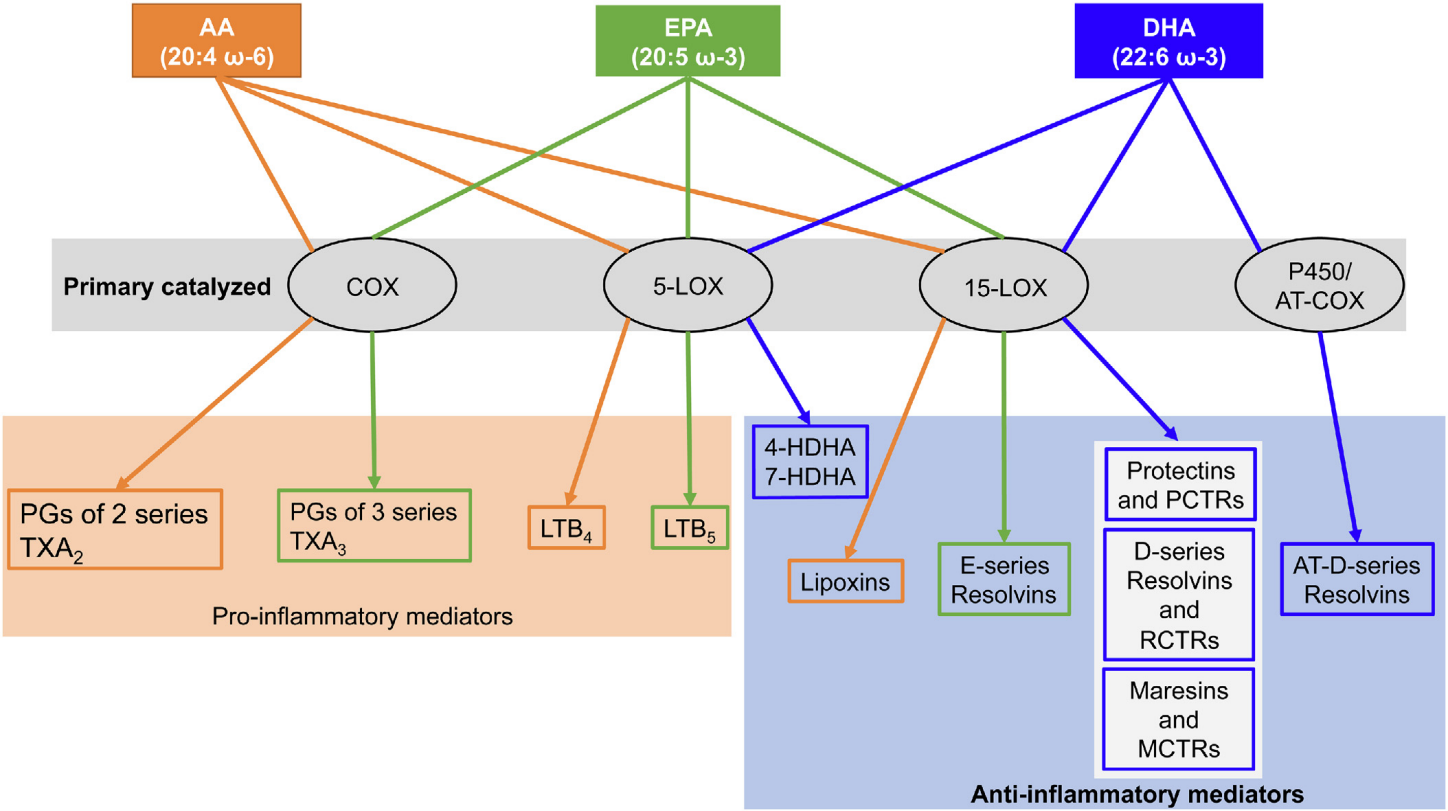
Lipid mediators derived from the three most abundant essential fatty acids, AA, EPA, and DHA, esterified in the sn-2 position of the phospholipids. Depending on the primary catalyzing enzyme, COX-2, and 5- and 15-LOXs, there is synthesis of a variety of bioactive lipids involved in inflammation as well as in resolution of the inflammatory response. Mediators from AA are highlighted in orange, EPA in green, and DHA in blue.

Figure 3 shows a scheme of bioactive lipids resulting from AA, EPA, and DHA. While many AA lipid mediators, as well as some EPA lipid mediators, have strong pro-inflammatory properties, all known DHA mediators (the docosanoids) act to protect and resolve inflammation (42, 43). They constitute part of a family named specialized pro-resolvin mediators that includes NPD1 and other protectins, maresins, and resolvins of the D series (43) and the newer sulfide conjugates of protectins (PCTRs), maresins (MCTRs), and resolvins (RCTRs). The synthetic mechanism to produce the specialized pro-resolvin mediators involves LOXs (including 15-LOX as primary catalyzer and 5-LOX as secondary catalyzer), COX (in the presence of aspirin), and cytochrome P450 enzymes (44). Information about the signaling mechanisms of DHA lipid mediators is still limited, especially identification of their receptors (Table 1). Most of the known receptors belong to the family of G protein-coupled receptors. In addition, some docosanoids share the same receptor, but their activation exerts specific biological activities (43).

**Table 1:** List of reported receptors of docosanoids

Name	Receptors	References	Expression in the Cornea
Resolvin D1	ALX/FPR2, GPR32 (DRV1)	(44)	Yes
Resolvin D2	GPR18 (DRV2)	(78)	ND
Resolvin D3	ALX/FPR2, GPR32 (DRV1)	(79)	Yes
Resolvin D4	N/A	—	—
Resolvin D5	GPR32	(80)	ND
Resolvin D6	N/A	—	—
Neuroprotectin D1	GPR37 (Pael-R)	(81)	ND
Maresin 1	LGR6	(82)	ND
Maresin 2	N/A	—	—

ND, not determined; N/A, not available.

**Fig. 4:**
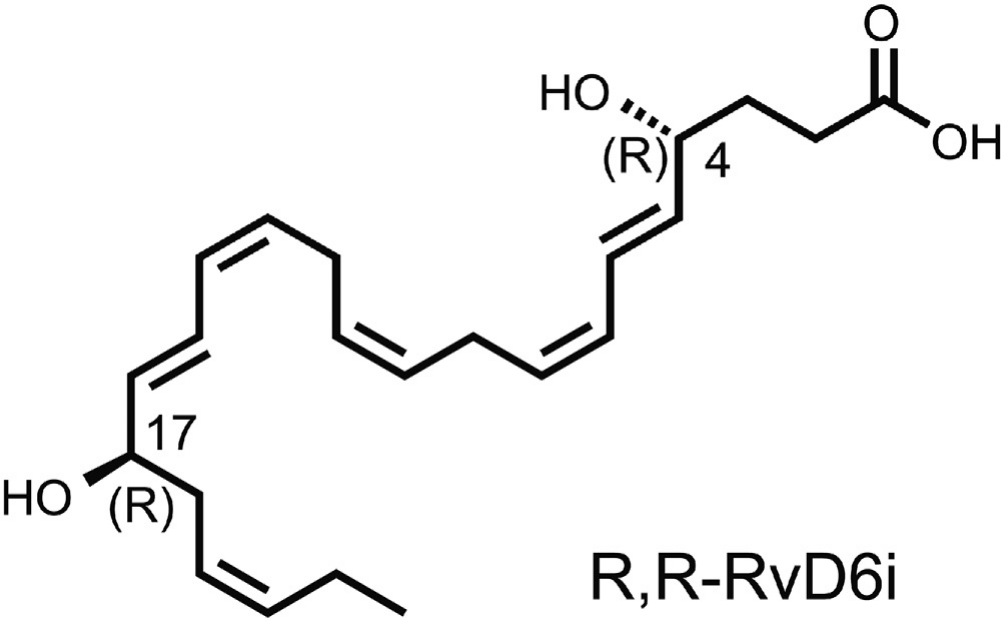
Structure of the RvD6i. The new isomer was synthesized after topical stimulation of mouse injured corneas with PEDF+DHA and released in tears. It was analyzed by LC-MS/MS and showed at least six matched daughter ions with an RvD6 standard but with an earlier retention time (40). Posterior studies show that the peak retention time coincides with chemically synthetized R,R-RvD6i in a chiral column (unpublished observations).

Recently, we discovered a novel docosanoid, a stereoisomer of resolvin D6 (RvD6), named RvD6i (Fig. 4), that is released in mouse tears after injury and treatment with PEDF+DHA (40). The fragmentation pattern of this new lipid shows at least six matched product ions that coincide with RvD6. Resolvin D6 had been found in some tissues, and studies in plasma from healthy individuals showed that RvD6 is a biomarker that decreases with aging (45). RvD6 is also released from stem cells isolated from human periodontal ligaments, which is important in tissue regeneration (46). However, RvD6 is not detected in normal human tears (47). Compared with treatments with PEDF+DHA and RvD6, the new RvD6i accelerated corneal wound healing and sensitivity, demonstrating a higher bioactivity (Fig. 4A, B).

## Use of DHA for DED

DED affects between 5% and 40% of adults older than 40 years (48, 49) with an estimated 16.4 million people impacted in the United States (50). In a recent Dry Eye Workshop (DEWS II), DE was defined as “a multifactorial disease of the ocular surface characterized by a loss of homeostasis of the tearfilm, and accompanied by ocular symptoms, in which tearfilm instability and hyperosmolarity, ocular surface inflammation and damage, and neurosensory abnormalities play etiological roles.” (Ref. 49; p. 278).

Within the last decade, there have been a number of clinical trials of DED patients with different etiologies using ω-3 fatty acid, DHA and EPA, supplementation with the argument that dietary fatty acids can be incorporated in the lacrimal gland as well as in plasma phospholipids (51). However, the effect of oral PUFA supplementation in DED is controversial. While some studies showed improvement, others showed insignificant effects. In Table 2, we summarized clinical trials conducted in the last 10 years in which supplementation with DHA was used to treat DED of different etiologies.

**Table 2:** Summary of clinical trials in the last 10 years for DED using ω-3 fatty acid treatment

Study	Number of Patients/Treatment	Randomized/Controlled	Masking	Effect	Comments
Brignole- Baudouin et al., 2011 (83)	DE, n = 127, time = 90 days Group 1, n = 61 142.5 mg EPA, 95 mg DHA, and supplements, three times daily Group 2, n = 66 Placebo, medium-chain triglyceride, three times daily	Yes/Yes	Double	No significant effect	Only decrease in the percentage of HLA-DR-positive cell was detected in treated group
Wojtowicz et al., 2011 (84)	DE, n = 36, time = 90 days Group 1 450 mg EPA, 300 mg DHA, and 1,000 mg flaxseed oil, one time daily Group 2 Placebo, wheat germ oil	Yes/Yes	Double	No significant effect	No changes in aqueous tear evaporation
Bhargava et al., 2013 (85)	DE, n = 528, time = 3 months Group 1, n = 264 325 mg EPA and 175 mg DHA, two times daily Group 2, n = 254 Placebo, two times daily	Yes/Yes	Double	Improved	—
Kangari et al., 2013 (86)	DE, n = 64, time = 30 days Group 1, n = 33 180 mg EPA and 120 mg DHA, two times daily Group 2, n = 31 Placebo, medium-chain triglyceride	Yes	Double	Slightly improved	—
Oleñik et al., 2013 (87)	Meibomian gland dysfunction, n = 64, time = 3 months Group 1, n = 33 Brudysec (350 mg DHA, 42,5 mg EPA, 30 mg DPA), three times daily Group 2, n = 31 Placebo, 500 mg sunflower oil, three times daily. All patients received cleaning the lid margins with neutral baby shampoo and artificial tears without preservatives	Yes/No	Double	Slightly improved	No significant differences in corneal staining from placebo
Ong et al., 2013 (88)	Healthy PRK patients, n = 18, time = 6 weeks Group 1, n = 9 250 mg EPA and DHA, 333 mg flaxseed oil, and 61 IU vitamin E, three times daily Group 2, n = 9 Control	Yes/Yes	Single	Improved	Treatment was 2 weeks before PRK surgery through 1 month after surgery
Sheppard et al., 2013 (89)	Post-menopausal women with DE, n = 38, time = 6 months Group 1, n = 19 49 mg ALA, 31.5 mg EPA, 3.75 mg DPA, 25 mg DHA, 177.5 mg LA, 60 mg GLA, <0.75 AA, and supplements, four times daily Group 2, n = 19 Placebo	Yes/Yes	Double	Improved	Placebo treatment also increased HLA-DR intensity by 36 ± 9% and CD11c by 34 ± 7% when compared with supplement treatment
Oleñik, 2014 (90)	DE, n = 905, time = 12 weeks Brudysec (350 mg DHA, 42,5 mg EPA, 30 mg DPA), three times daily. No control of placebo	No/No	No	Improved	A total of 68.1% of patients reported better tolerance to contact lenses after treatment
Georgakopoulos et al., 2017 (91)	Diabetic patients with DE, time = 3 months Group 1, n = 36 170 mg EPA and 115 mg DHA, three times daily	No/No	No	Improved	—
Bhargava et al., 2015 (92)	Computer-related DE, n = 456, time = 3 months Group 1, n = 220 180 mg EPA and 120 mg DHA, two times daily Group 2, n = 236 Placebo containing olive oil, two times daily Baseline (T0), 1 month of treatment (T1), 2 months of treatment (T2), 3 months of treatment (T3)	Yes/Yes	Double	Improved	—
Deinema et al., 2017 (56)	DE, n = 54, time = 3 months Group 1, n = 18 Krill oil (945 mg/day EPA + 510 mg/day DHA) Group 2, n = 19 Fish oil (1,000 mg/day EPA + 500 mg/day DHA) Group 3, n = 17 Placebo (olive oil, 1,500 mg/day)	Yes/Yes	Double	Slightly improved	Both krill and fish oil moderately reduced the DE symptoms. The pro-inflammatory cytokine, interleukin 17A, was significantly reduced in the krill oil group only at day 90
Goyal et al., 2017 (93)	LASIK patients, n = 60, time = 12 weeks Group 1, n = 30 180 mg EPA and 120 mg DHA, four times daily Group 2, n = 3 Placebo	Yes/Yes	Double	Slightly improved	Fewer eyes had conjunctival staining with Lissamine
DREAM, 2019 (52)	DE, n = 499, time = 12 months Group 1, n = 329 400 mg EPA and 200 mg DHA, five times daily Group 2, n = 170 Placebo, 1,000 mg refined olive oil, five times daily	Yes/Yes	Double	No significant effect	Significantly increased EPA and DHA in red blood cells
**Fogt et al., 2019** (58)	**DE, n = 19, time = 1 h** **Drug 1, n = 19** **Refresh Optive plus Omega-3, flaxseed oil** **Drug 2, n = 19** **Refresh Optive** **The drug is randomly picked for two different visits (>2 days between)**	**Yes/Yes**	**Double**	**Improved (short time)**	**The lipid layer thickness (LLT) was increased from baseline at 15 min for both treatments. Only Refresh Optive plus Omega-3 patients had higher LLT up to 1 h after instillation**

Bold type indicates the clinical trial using topical eye drops. LASIK, laser in situ keratomileusis.

One of the most important trials, the DREAM study, which involved a total of 499 patients with 329 receiving 12 months of supplementation with EPA and DHA and 170 patients treated with refined olive oil as a placebo (52), suggested that there was no improvement. This study increases the doubtfulness about the benefit of DHA in the treatment of DED. For this reason, in this review, we point out problems that may explain the controversial results of DHA supplementation.

One concern is the form of DHA supplementation. Most of the studies employed natural enriched fish oil. However, analysis of fish oil composition showed that the PUFAs are mainly esterified in triglycerides. DHA from the diet needs to be taken up by the liver before being esterified in the sn-2 position of membrane phospholipid, mainly PC (53). DHA-phospholipids are then packaged in VLDLs or other lipoproteins before being released into the blood stream (53, 54). Therefore, the possibility that supplementation of DHA or EPA from fish oil reaches the ocular surface, especially the cornea, is very low. This is supported by previous studies where krill oil, which mainly contains PC with long chain PUFAs, showed a higher absorption rate in rat blood and brain than fish oil (55). There is only one study that uses krill oil to treat DED, a small clinical trial (18 participants per group) in which Deinema and colleagues showed lower Ocular Surface Disease Index and IL-17A levels in krill oil supplementation than in fish oil after 90 days of treatment (56) and Table 2.

In addition, it is important to note that the cornea is avascular, therefore, the probability that dietary fatty acids are incorporated into the corneal cellular membrane is limited. This is supported by a study using ^14^C-labeled DHA given orally to rats, which showed a very small amount (less than 0.03% of the oral dose) of DHA that reached the eye compartment (57). Of this quantity, the amount that might get into the cornea is very low because the retina takes most of the DHA from subretinal blood vessels. Therefore, PUFA enrichment in the lacrimal gland is insufficient to ensure a beneficial treatment in the cornea.

To our knowledge, there is only one clinical trial using topical DHA (58) (Table 2). This trial was based on previous studies showing that AA, DHA, and EPA were found in the tears of patients with DED and that the ratio of ω-6 (AA):ω-3 (DHA+EPA) correlates with the severity of the tear film dysfunction (59). The small trial (19 patients treated topically with DHA) demonstrated that treatment with eye drops containing omega-3 fatty acids increases lipid layer thickness of the tear film up to 1 h after instillation (58).

**Fig. 5:**
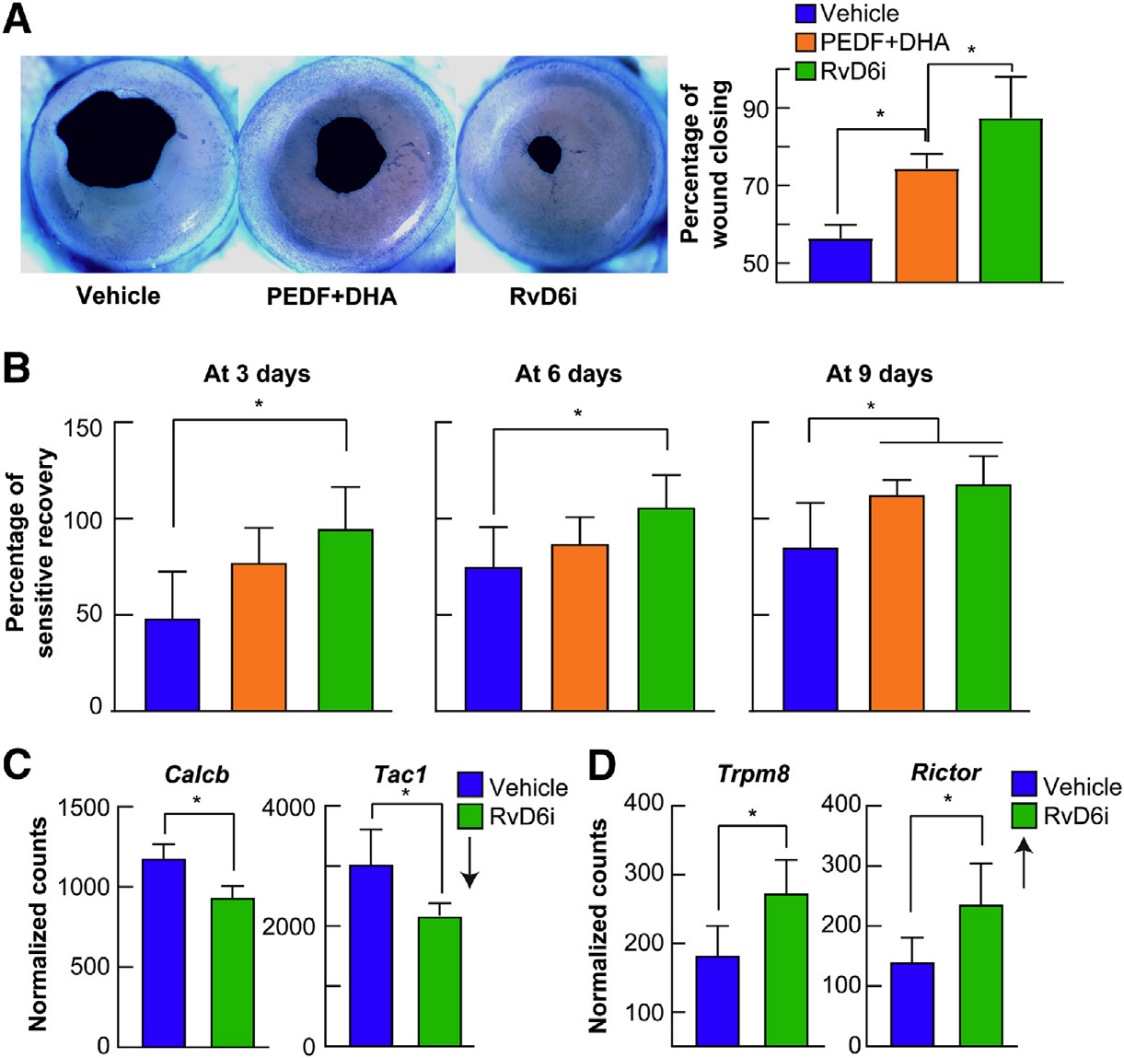
RvD6i accelerates corneal wound healing and sensitivity. A: Representative images of mouse cornea wounded area stained with methylene blue after 20 h of an injury that damaged the epithelial and anterior stroma nerves. The animals received eye drops containing PEDF+DHA or RvD6i in similar concentrations three times per day. The images were taken with a dissecting microscope and quantified using Photoshop software (40). B: Recovery of cornea sensitivity at 3, 6, and 9 days after injury and treatment with PEDF+DHA or RvD6i (three times per day) using a noncontact aesthesiometer. RvD6i-treated mice recovered sensitivity sooner than PEDF+DHA-treated corneas. C: Expression of genes involved in inflammation and pain in the TG of RvD6i topically treated corneas. TG were obtained 12 days after cornea injury and treatment with RvD6i and analyzed by RNA sequencing (40). *Calcb* and *Tac1* genes were downregulated while *Trpm8* and *Rictor* genes were upregulated in the TG neurons by cornea treatment with RvD6i. ∗*P* < 0.05 with the *t* test statistical analysis to compare two groups at 95% of the confidence level.

Lastly, our animal studies show that DHA is rapidly incorporated in the corneal phospholipids, mainly in PE and PC, to increase nerve density. A decrease in nerve density is a well-documented alteration in DED that requires both PEDF and DHA to regenerate the nerves. The treatment releases DHA and stimulates the synthesis of RvD6i, and this docosanoid increases wound healing and sensitivity (Fig. 5A, B) and could be of better therapeutic use than DHA for DED (40).

The effectiveness of docosanoids in decreasing inflammation and increasing corneal wound healing, nerve regeneration, and tear secretion has been demonstrated clearly on several different models of injury, infection, diabetes, corneal angiogenesis, and transplantation (Table 3). These results emphasized the action of docosanoids as potent drugs.

**Table 3:** In vivo studies using PEDF+DHA or docosanoids for corneal damage

Animal	Model	Docosanoids	Administration	Key Result
Mouse	Corneal epithelium removal up to the corneal/limbal border (94)	NPD1, 17S-HDHA	Topical eye drops, three times daily for 96 h	Increased the rate of re-epithelialization. Increased PMNs in the cornea. Decreased formation of the pro-inflammatory chemokine KC
Mouse	Suture-induced inflammatory corneal angiogenesis (95)	RvD1	Subconjunctival injection every 48 h; time = 14 days	Reduced numbers of infiltrating neutrophils and macrophages and reduced mRNA expression levels of TNF-α, IL-1α, IL-1β, VEGF-A, VEGF-C, and VEGFR2. Suppressed suture-induced or IL-1β-induced hemangiogenesis but not lymphangiogenesis
**Rabbit**	**Experimental PRK** (19, 20, 21)	**PEDF+DHA, PEDF domains + DHA**	**Topical using collagen shield, twice a week; time = 8 weeks**	**Increased nerve density and tear secretion in treated group for 8 weeks. with PEDF+DHA. NPD1 synthesis peaked at 1 week and was four times higher in the PEDF+DHA-treated group than in the controls. The 44-mer domain of PEDF is more potent than the 34-mer domain**
**Rabbit**	**Experimental PRK** (41)	**NPD1**	**Topical eye drops of NPD1 (33 ng/eye) three times daily for 6 weeks**	**Increased subepithelial corneal nerves and tear secretion. Decreased neutrophil infiltration after 2 and 4 days of treatment**
Mouse	Corneal allotransplantation (96)	RvD1 analog	Intravenous injection	Reduced allosensitization. Reduced angiogenesis at the graft site. Enhanced graft survival
Mouse	Type 2 diabetes (97)	RvD1, RvD1-methyl ester, RvD2-methyl ester	Daily intraperitoneal injections of 1 ng/g body weight for 8 weeks	Reduced the diabetes-induced corneal nerve lost. Methyl ester version is less bioactive than free fatty acid
**Rabbit**	**HSV1 corneal infection** (22)	**PEDF+DHA**	**Topical eye drops, three times daily for 2 weeks. Topical using collagen shield, twice a week for 10 weeks more**	**Stronger infiltration of CD4+T cells, neutrophils, and macrophages at 7 days, then decreased by 14 days. Corneal nerve density increased at 12 weeks with functional recovery of corneal sensation**
**Mouse**	**Type 1 diabetes. Corneal epithelium removal inside 2 mm diameter central area** (23)	**PEDF+DHA**	**Topical eye drops, three times daily for 14 days**	**Increase in corneal epithelial nerve regeneration, SP-positive nerve density and tear volume. Accelerated corneal wound healing, selectively recruited type 2 macrophages, and prevented neutrophil infiltration**
**Mouse**	**Corneal nerve cutting** (27)	**PEDF+DHA**	**Topical eye drops, three times daily for 7 days**	**Increased nerve regeneration and tear secretion. Phospholipase A2 activity of the PEDF-R is required for the working mechanism**
Mouse	Type 1 diabetes. Corneal epithelium removal inside 2 mm diameter central area (98)	RvD1	Topical eye drops, four times daily for 14 days	Promotes corneal epithelial wound healing and nerve regeneration
**Mouse**	**Corneal epithelium removal inside 2 mm diameter central area** (40)	**RvD6i**	**Topical eye drops, three times daily for 12 days**	**Discovered the RvD6i underlying the mechanism of PEDF+DHA. Increased corneal wound healing, sensitivity, and nerve regeneration. Reduced inflammatory- and pain-related neuropeptides, increased ion channel gene expression in TG**

Bold type indicates studies from our laboratory. HSV1, herpes simplex virus.

## RvD6i regulates genes involved in neurogenesis and pain in the TG

Previous studies have showed that cornea treatment with PEDF and DHA also stimulated the synthesis of the docosanoid NPD1. However, the synthetized amount is much lower than RvD6i (19, 40). When adding NPD1 to injured corneas, there is an increase in the gene expression and protein levels of the neurotrophins NGF, brain-derived neurotrophic factor (BDNF), and semaphorin 7A (Sema7A) that stimulate axon growth (27). These proteins are secreted into tears and activate receptors in the corneal nerve terminals to facilitate downstream signaling as well as retrograde to the neurons of the TG.

Using RNA-sequencing to analyze the gene expression in TG from the injured corneas of mice, we reveal that the product of PEDF+DHA, RvD6i, applied topically to the cornea induces the expression of two interesting genes in the TG, chromosome 9 open reading frame 72 (*C9orf72*) and glycoprotein M6A (*Gpm6A*) (40). These genes stimulate neurogenesis and growth cone formation (60, 61).

Ocular pathologies that damage corneal nerves in many cases produce neuropathic pain (62). In addition, there are a significant number of patients who have symptoms of DED and experience neuropathic pain, suggesting that there is an active cornea-TG relationship (63). Two genes involved in pain were decreased in corneas treated with RvD6i: *Tac1* that encodes substance P (SP), which is one of the most abundant neuropeptides expressed in corneal nerves (4, 64, 65), and *Calcb*, which encodes calcitonin gene-related peptide (also abundant in corneal nerves) (4, 20) (Fig. 5C). Both neuropeptides have important roles in neurogenic inflammation and pain (66, 67). In addition, corneal treatment with RvD6i increased the gene expression of transient receptor potential melastatin 8 (*Trpm8*) (Fig. 5D). TRPM8 ion channels are cool sensors that regulate the wetting of the ocular surface and produce an analgesic effect on chronic pain (68, 69, 70, 71, 72). Our prior studies in a mouse model where the nerves had been damaged at the level of the anterior stroma, showed that cornea TRPM8-positive nerve fibers only reach 50% of their normal density after 3 months of injury, suggesting that the decrease in TRPM8 may contribute to DE-like pain (73). Therefore, decreased expression of SP and calcitonin gene-related peptide and increased expression of TRPM8 after injury and treatment with RvD6i suggests that the new docosanoid could protect corneas from pain. It also opens avenues of potential therapeutic exploration for ocular surface damage, especially corneal neurotrophic ulcers, because previous studies have shown ocular pain as a side effect of increased corneal nerve regeneration caused by topical treatment with NGF (74). Previous studies using RvD1 and RvD5 had shown pain attenuation in a mouse model of tibia bone fracture, while RvD3 and RvD4 had no effect (75). These differences could be due to different expression of its receptors. In an osteoporosis mouse model, the precursor of RvDs, 17R-HDHA, decreases pain behavior probably through activation of AXL receptors (76). Another important finding is that RvD6i is a strong inducer of the gene expression of *Rictor* in the TG (40) (Fig. 5D). RICTOR is a key component of the mammalian target of rapamacyn-insensitive complex 2 (mTORC2) and plays a role in anti-inflammation and axon growth of sensory neurons after injury (77).

**Fig. 6:**
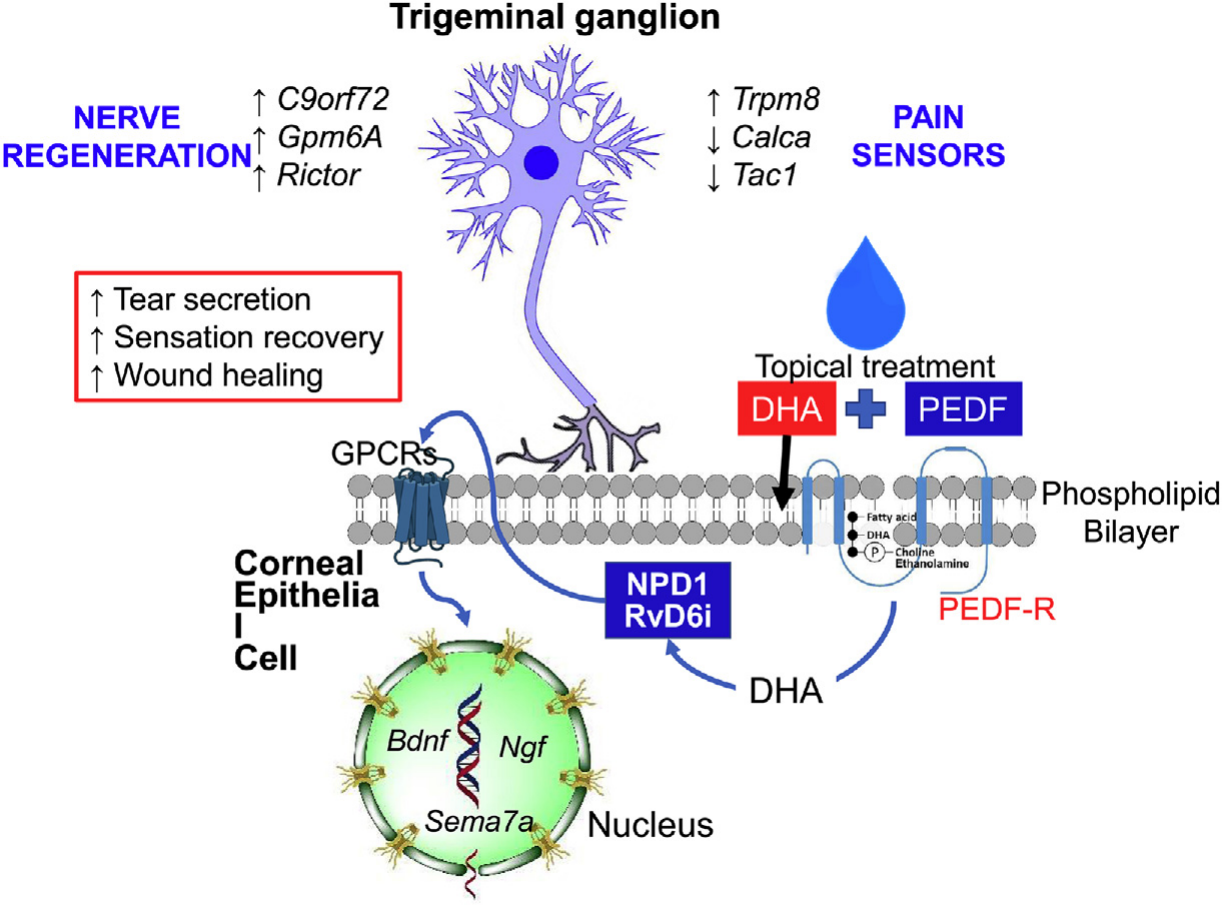
Schematic model of signaling stimulated by the combination of PEDF+DHA. DHA is rapidly incorporated into membrane phospholipids from corneal epithelium and then released after stimulation by PEDF of the PEDF-R with calcium-independent phospholipase A2 (iPLA2ζ) activity. Free DHA is then the substrate for docosanoids such as NPD1 and the novel RvD6i. These docosanoids are then released into tears and, by autocrine stimulation, to an undefined GPRC receptor(s) that induces the gene and protein expression of neurotrophic factors NGF, BDNF, and semaphorin 7A (Sema7A) that are secreted into tears and enhance axon outgrowth. RvD6i stimulates corneal wound healing, corneal sensation and nerve recovery, and tear secretion. The mechanism involves changes in the TG transcriptome with activation of genes related to neurogenesis and modulation of genes implicated in neuropathic pain. Treatment with PEDF or DHA alone does not activate these pathways, and therefore, there was no increase in cornea nerve regeneration (19). Gpm6A, glycoprotein M6A; C9orf72, chromosome 9 open reading frame 72; Trpm8, transient receptor potential melastatin 8.

A summarized scheme of the signaling pathways of docosanoids stimulated by PEDF and DHA is shown in Fig. 6.

## Conclusions

Cornea innervation plays a pivotal role in maintaining the homeostasis of the ocular surface and tissue clarity (7). Damage to corneal nerves produces a decrease in tear production and blinking reflex and can impair epithelial wound healing resulting in loss of transparency and vision (10, 11, 12, 13, 8, 9). Therefore, better knowledge on corneal nerve function and repair will increase therapeutic strategies for pathologies that affect corneal innervation. DHA-derived docosanoids, such as the new mediator RvD6i, could serve as potential treatment options to reduce cornea-related inflammation. The effect of this lipid in accelerating nerve regeneration and modulating the gene expression of components of neuropathic pain in the TG could provide a new alternative in the treatment of patients with DE following refractive surgery as well as possible cotreatment to several pathologies that decrease corneal nerve density. Prospective human clinical trials will be needed to confirm optimal dosing, modes of administration, efficacy, and safety of these promising new treatments for DE and ocular surface diseases.

## Conflict of interest

The authors declare that they have no conflicts of interest with the contents of this article.
